# PD-1 and LAG-3 were optimal combination of immune checkpoints for predicting poor clinical outcomes of patients with ovarian cancer

**DOI:** 10.3389/fimmu.2025.1656242

**Published:** 2025-08-14

**Authors:** Yifan Wu, Cunte Chen, Yingwen Cui, Ruoyao Zou, Yaoxiang Yang, Fengjie Sun, Yanzi Du, Peipei Wang

**Affiliations:** ^1^ Center for Reproductive Medicine, Ren Ji Hospital, School of Medicine, Shanghai Jiao Tong University, Shanghai, China; ^2^ Guangzhou First People's Hospital, South China University of Technology, Guangzhou, China; ^3^ Guangzhou Women and Children’s Medical Center, Guangzhou Medical University, Guangzhou, China; ^4^ Department of Neonatology, the Fifth Affiliated Hospital of Guangzhou Medical University, Guangzhou, China

**Keywords:** immune checkpoint, PD-1, LAG-3, prognosis, ovarian cancer

## Abstract

**Background:**

Although immune checkpoint blockade (ICB) therapy has transformed the therapeutic landscape for ovarian cancer (OC), the predictive utility of immune checkpoint (IC) expression signatures in stratifying clinical outcomes requires further systematic interrogation.

**Methods:**

Transcriptomic profiles from 147 OC patients within The Cancer Genome Atlas (TCGA) cohort were interrogated to assess the prognostic significance of ICs. These genomic findings were subsequently validated through immunohistochemical analysis of an independent institutional cohort comprising 74 OC tissue specimens.

**Results:**

Both TCGA and validation cohorts demonstrated that elevated expression of PD-1 and LAG-3 correlated with inferior overall survival (OS) in patients with OC. Importantly, among the ICs, PD-1/LAG-3 co-expression emerged as the optimal combinatorial biomarker, independently predicting adverse outcomes [hazard ratio (HR) = 1.74, 95% confidence interval (CI): 1.12-2.70, *P* < 0.001]. The derived nomogram model incorporating PD-1/LAG3 status, TNM stage, histologic grade, and age generated patient-tailored 1–5 year OS rate estimates. Notably, risk stratification using this model significantly enhanced prognostic precision versus conventional parameters (TNM stage or histologic grade) alone, especially in patients with serous cystadenocarcinoma.

**Conclusion:**

Elevated IC expression correlated with poor OS in OC patients. Specifically, PD-1/LAG-3 co-expression emerged as the optimal prognostic biomarker pair, representing a promising therapeutic target for dual checkpoint blockade strategies in OC.

## Introduction

Ovarian cancer (OC) is a highly aggressive malignancy and remains one of the leading causes of cancer-related deaths among women worldwide (1, 2). A significant number of patients are diagnosed at advanced stages, and despite aggressive treatments such as surgery and platinum-based chemotherapy, a large proportion of patients succumb to the disease due to disease progression (3). The 5-year survival rate for ovarian cancer remains below 50% (4). The current standard of care involves tumor debulking surgery followed by platinumbased chemotherapy. While this combined therapeutic approach has led to some improvement in overall survival rates, over 70% of patients experience recurrence within five years (4). Even more concerning is the absence of reliable biomarkers that can accurately predict prognosis in ovarian cancer, which complicates clinical decision-making and patient management.

In recent years, the tumor microenvironment (TME) has emerged as a critical player in tumor initiation, progression, metastasis, and immune evasion (2, 5). The TME can be viewed as a “complicit ecosystem,” consisting of cancer cells, stromal cells, immune cells, and extracellular matrix components. These elements interact to create a “nurturing environment” that supports tumor growth while simultaneously impeding the normal function of the immune system (6). Among the various components of the TME, T cells play a pivotal role, not only in tumor progression but also in immune suppression (2, 5). T cell exhaustion, characterized by the loss of effector functions and the persistent expression of inhibitory receptors, has become a major focus of research (2, 7, 8). Understanding how to regulate this exhaustion state remains a critical challenge in cancer immunotherapy.

The underlying mechanism of cancer immunotherapy involves activating the host immune system to mount an active or passive immune response against tumor cells (9). Immunotherapies have shown remarkable efficacy in several malignancies, including melanoma, renal cell carcinoma, and lung cancer (10–12). With the substantial progress made in immunotherapy across various cancers, the field of ovarian cancer immunotherapy has attracted increasing attention. Notably, immune checkpoint blockade (ICB) therapies, targeting the PD-L1/PD-1 axis, have demonstrated significant success in several cancer types, offering new hope for ovarian cancer treatment (2, 13, 14). However, the clinical reality remains less promising, as ovarian cancer patients exhibit poor responses to ICBs (15, 16). This is primarily due to the highly immunosuppressive characteristics of the ovarian cancer TME, which often lacks sufficient infiltration of tumor-targeting immune cells, especially CD8+ T cells and activated CD4+ T cells (16, 17). These factors contribute to the limited efficacy of immunotherapy in OC (8). Additionally, ovarian cancer TME also exhibits low PD-L1 expression and weak immunogenicity, further restricting the effectiveness of immunotherapies (2, 8). Therefore, a deeper understanding of the unique immune characteristics of the TME in OC, particularly the expression of IC molecules and their association with prognosis, is essential for identifying new immune targets.

In this study, we reveal that the co-expression of LAG-3 (Lymphocyteactivation gene 3) and PD-1 in ovarian cancer patients represents a novel and promising biomarker for predicting patient prognosis. Our findings indicate that the simultaneous expression of these two immune checkpoints (ICs) is associated with poor survival outcomes, underscoring their potential as prognostic indicators. These findings provide a new perspective on personalized treatment strategies for ovarian cancer, emphasizing the importance of combination therapy targeting both LAG3 and PD-1.

## Materials and methods TCGA dataset

RNA sequencing data from 147 treatment-naïve OC patients with complete clinical information were retrieved from The Cancer Genome Atlas (TCGA) dataset via International Cancer Genome Consortium (ICGC) in the UCSC Xena platform (https://xenabrowser.net/datapages/) and designated as the training cohort (7, 18). Clinicopathological characteristics—including age, primary tumor, regional lymph node, distant metastasis (TNM) stage, anatomic subdivision, histologic grade, histologic type, survival time and vital status— were extracted (**Supplementary Table S1**). As this study utilized publicly available, de-identified TCGA data, institutional review board approval was waived.

### OC tissue samples

The validation cohort comprised formalin-fixed paraffin-embedded (FFPE) tumor specimens obtained from 74 treatment-naïve OC patients treated at our clinical center (November 2017 - April 2024). All specimens were acquired during diagnostic biopsy or surgical resection. The enrolled OC patients meeting strict criteria: (1) histopathologically confirmed; (2) treatment-naïve; (3) available FFPE blocks with ≥70% tumor content; (4) complete International Federation o Gynecology and Obstetrics (FIGO) staging/survival documentation. Exclusion criteria: prior therapies or inadequate clinical data. Patients were staged according to FIGO guidelines. Clinicopathological parameters are detailed in **Supplementary Table S1**. Final follow-up occurred on 20 June 2025, yielding a median observation period of 51.9 months (range: 14.7-91.6) for surviving patients. The institutional review board of Guangzhou Women and Children’s Medical Center approved this study (No. 2024-299A01), with written informed consent obtained from all participants in compliance with Helsinki Declaration principles.

### Immunohistochemistry

Four-micron sections from FFPE tumor specimens underwent sequential processing: deparaffinization in xylene, rehydration through graded ethanol series, and heat-induced epitope retrieval in 1 mM EDTA buffer (pH 8.0) using microwave irradiation. Endogenous peroxidases were quenched with 0.3% H_2_O_2_, followed by blocking of nonspecific sites with 5% goat serum. Primary antibody incubations employed rabbit anti-human PD-1 (Abcam ab237728; 1:200) and LAG-3 (Abcam ab209236; 1:200). Detection utilized HRPconjugated secondary antibodies (DAKO EnVision™) with DAB chromogenic development. Counterstaining with hematoxylin preceded dehydration and coverslipping. Immunoreactivity was quantified in five randomly selected highpower fields (400×) by two independent pathologists. Scoring criteria integrated intensity and distribution: 0: <5% positive cells; 1+: weak staining in 5-25% cells; 2+: moderate staining in 25-50% cells; 3+: strong staining in >50% cells. The histoscore (H-score) was calculated as: H-score = Σ (intensity grade × % positive cells) (7).

### Construction of nomogram model

Using R statistical environment (v4.3.2; https://www.r-project.org/), data preprocessing was performed with the foreign package (v0.8-85) for format conversion. The nomogram model for predicting OS rate of OC patients was constructed via the rms package (v6.7-1) (7). According to the total risk scores obtained from the nomogram model, OC patients can be divided into three groups: favorable, intermediate and poor-risk using X-tile software (v3.6.1).

### Statistical analysis

Statistical analyses used R language (version 4.3.2, https://www.r-project.org/) and SPSS software (version 22.0, IBM, Armonk, NY, USA). IC biomarker thresholds were optimized using: (1) *survminer*’s survival-based partitioning (19); (2) X-tile’s outcome-driven minimization (18). Survival curves compared by log-rank test (20, 21). Spearman correlations assessed inter-marker associations. Categorical comparisons employed χ²/Fisher tests per Cochran’s rules. Area Under Curve (AUC) in the Receiver Operating Characteristic (ROC) was determined by “survivalROC”. Univariate and multivariate Cox proportional hazards models identified OS predictors. Significance: two-sided *P* < 0.05.

### Results OS analysis of ICs in patients with OC

**Figure 1:**
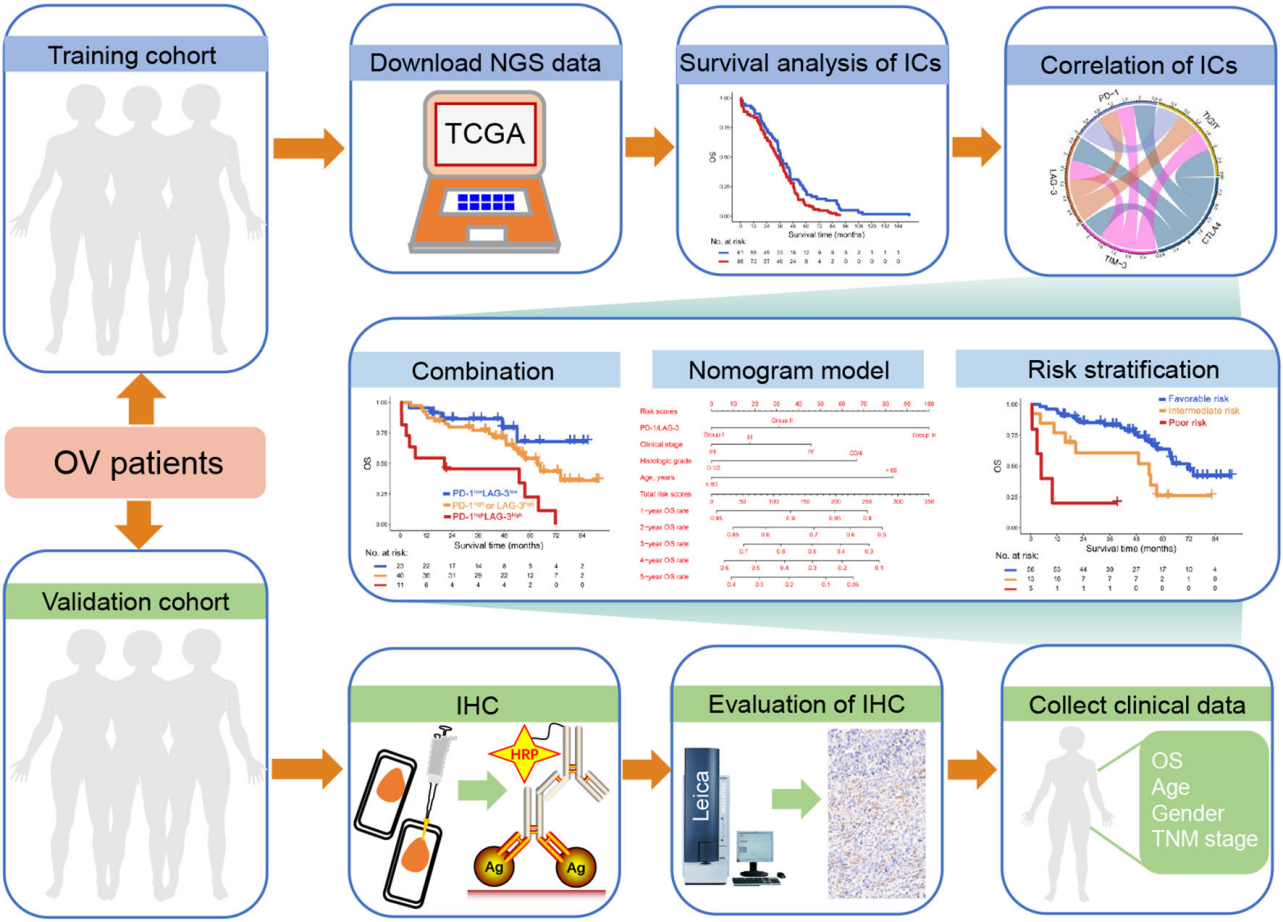
Study schematics. Next-generation sequencing (NGS) data from ovarian cancer (OC) patients within The Cancer Genome Atlas (TCGA) database and immunohistochemistry (IHC) data derived from our clinical center were designated as the training and validation cohorts, respectively. Initially, we assessed the association between immune checkpoint (IC) expression and overall survival (OS) in OC patients, alongside evaluating inter-IC correlations. Subsequently, the combinatorial prognostic impact of ICs on OC was investigated, and OS rates were visually represented using a nomogram model. Finally, a risk stratification system incorporating IC expression profiles and clinical parameters was constructed.

**Figure 2:**
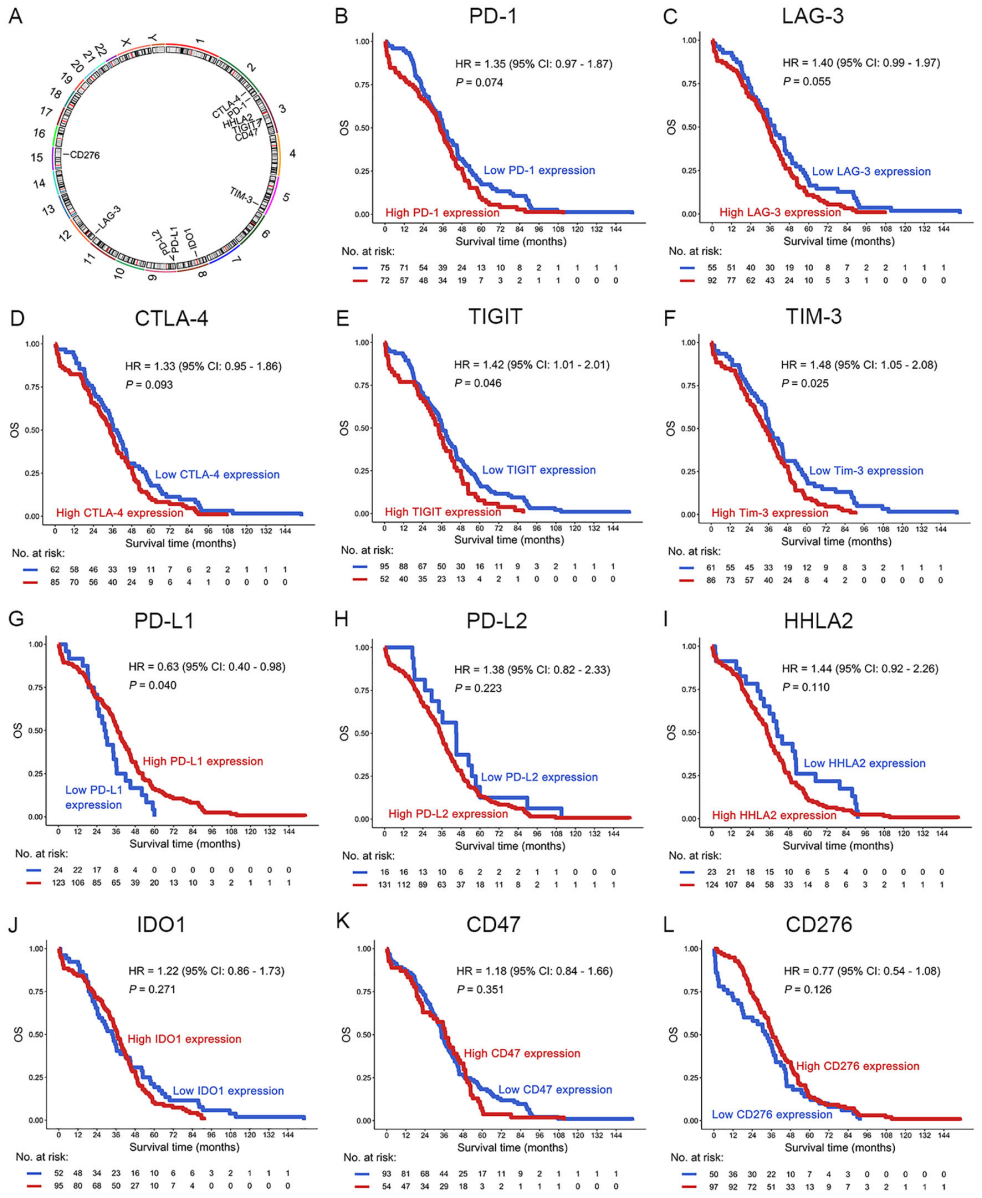
Prognostic analysis of ICs in OC patients in the TCGA dataset. **(A)** The location of the IC genes in the chromosomes. **(B–L)** OS analysis of PD-1 **(B)**, LAG-3 **(C)**, CTLA-4 **(D)**, TIGIT **(E)**, TIM-3 **(F)**, PD-L1 **(G)**, PD-L2 **(H)**, HHLA2 **(I)**, IDO1 **(J)**, CD47 **(K)** and CD276 **(L)** in OC patients. The optimal prognostic cutpoints for ICs were determined by maximally selected rank statistics in the “survminer” package. This is an outcome-oriented method that identifies a cutpoint that best separates survival outcomes.

To evaluate the prognostic significance of ICs in patients with OC, we first assessed associations between mRNA expression of 11 IC molecules (7, 18, 22) (PD-1, PD-L1, PD-L2, CTLA-4, LAG-3, TIM-3, TIGIT, HHLA2, CD47, IDO1, CD276) and OS using TCGA dataset (**Figures 1**, **2A**). Elevated mRNA expression of PD-1, LAG-3, and CTLA-4 demonstrated a non-significant trend toward adverse survival outcomes (*P* < 0.10; **Figures 2B–D**). Moreover, high TIGIT and TIM-3 expression significantly correlated with reduced OS (*P* < 0.05; **Figures 2E, F**). However, high PD-L1 expression was significantly correlated with long OS (*P* = 0.040; **Figure 2G**). No statistical associations were observed for PD-L2, HHLA2, IDO1, CD47, or CD276 (*P* > 0.10; **Figures 2H–L**). Consequently, PD-1, LAG-3, CTLA-4, TIGIT, and TIM-3 emerged as candidate prognostic biomarkers for subsequent analyses.

### PD-1 and LAG-3 were optimal combination of ICs for predicting poor OS in patients with OC

**Figure 3:**
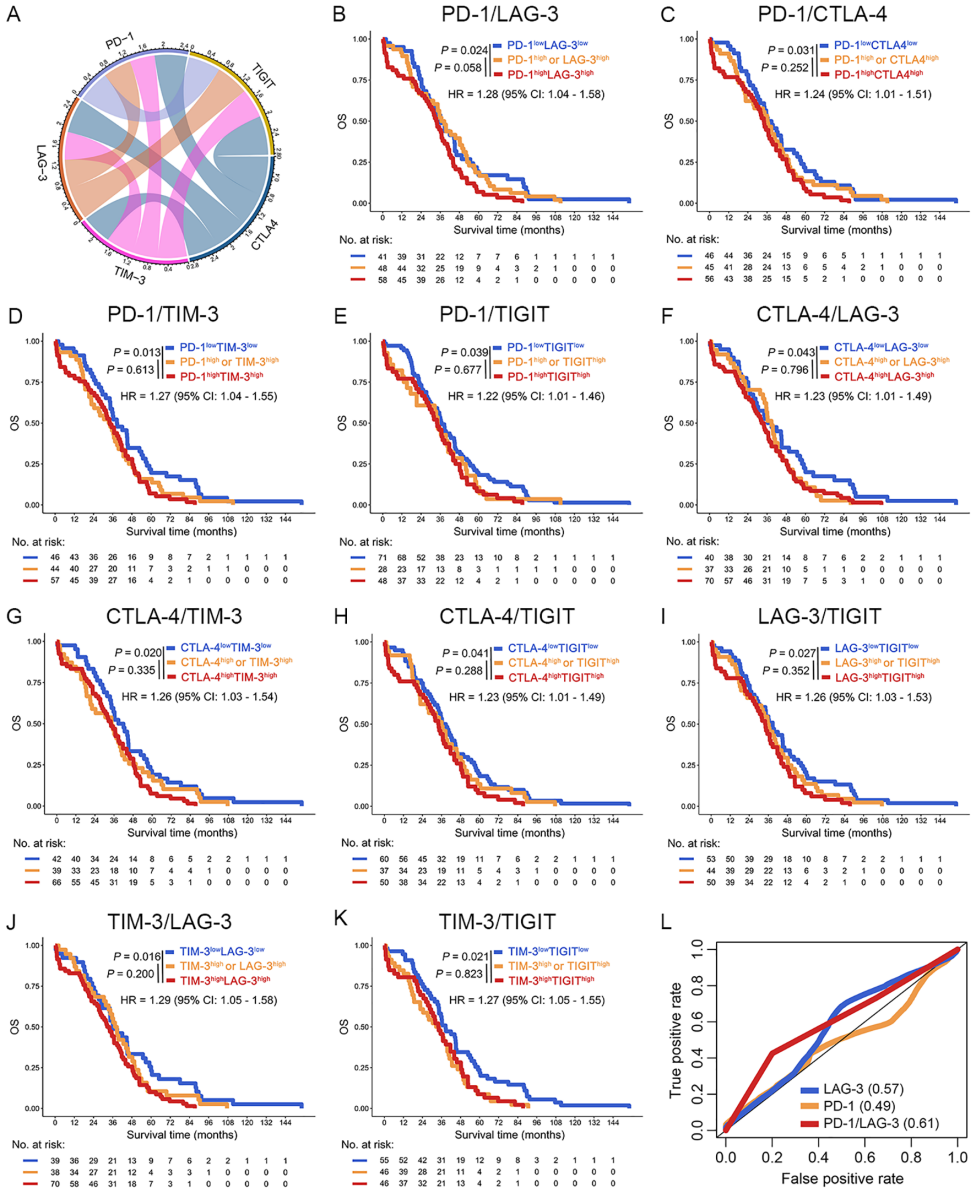
Increase co-expression of ICs predicted poor OS of OC patients in the TCGA dataset. **(A)** The chord diagram illustrated the co-expression network among PD-1, LAG-3, TIM-3, CTLA-4, and TIGIT. Connecting bands represented significant positive correlations between gene pairs, with band width proportional to the magnitude of the correlation coefficient (all *P* < 0.05). **(B–K)** Kaplan-Meier curves were plotted based on the different groups of PD-1/LAG-3 **(B)**, PD-1/CTLA-4 **(C)**, PD-1/TIM-4 **(D)**, PD-1/TIGIT **(E)**, CTLA-4/LAG3 **(F)**, CTLA-4/TIM-3 **(G)**, CTLA-4/TIGIT **(H)**, LAG-3/TIGIT **(I)**, TIM-3/LAG-3 **(J)** and TIM-3/TIGIT **(K)** in OC patients. **(L)** ROC curves were used to evaluated the optimal IC combination for predicting poor prognosis in OC patients.

Intercheckpoint relationships among PD-1, LAG-3, CTLA-4, PD-L1, TIGIT, and TIM-3 were quantified in the TCGA dataset. Significant co-expression patterns emerged (R > 0.52, *P* < 0.001; **Figure 3A**), aligning with known synergistic effects of combinatorial checkpoint blockade. Evaluation of IC (PD-1, LAG-3, CTLA-4, PD-L1, TIGIT, and TIM-3) pairs revealed each combination predicted poor OS in patients with OC (*P* < 0.05; **Figures 3B–K**). This systematic characterization informed subsequent optimization of multibiomarker prognostic signatures. Interestingly, among OC patients with high mRNA expression of PD-1 concomitant high mRNA expression of LAG-3 was associated with poor OS in the TCGA dataset [PD-1^high^LAG-3^high^ (group III) vs. PD-1^high^ or LAG-3^high^ (group II), *P* = 0.058; PD-1^high^LAG-3^high^ (group III) vs. PD1^low^LAG-3^low^ (group I), *P* = 0.024] (**Figure 3B**). Furthermore, increased coexpression of PD-1/LAG-3 was an independent predictor of poor OS in OC patients by univariate and multivariate Cox proportional hazards model [hazard ratio (HR) = 1.74, 95% confidence interval (CI): 1.12-2.70, *P* < 0.001; **Table 1**]. Notably, combinatorial overexpression of checkpoint pairs (PD-1/CTLA-4, PD-1/TIM-3, PD-1/TIGIT, CTLA-4/LAG-3, CTLA-4/TIM-3, CTLA-4/TIGIT, LAG3/TIGIT, TIM-3/LAG-3, TIM-3/TIGIT) failed to significantly improve OS prediction over single-checkpoint elevation in patients with OC (*P* > 0.05, **Figures 3C–K**). Accordingly, the superior predictive value of PD-1/LAG-3 co-expression was identified over single biomarkers or other combinations (**Figure 3L**).

**Table 1:** Univariate and multivariate Cox regression analysis in patients with ovarian cancer.

Variables*	Univariate Cox regression				Multivariate Cox regression			
	TCGA		Clinical center		TCGA		Clinical center	
	HR (95% CI)	*P*	HR (95% CI)	*P*	HR (95% CI)	*P*	HR (95% CI)	*P*
PD-1/LAG-3 (ref: Group I)								
Group II	1.11 (0.73-1.70)	0.626	1.95 (0.73-5.23)	0.185	1.18 (0.76-1.83)	0.467	2.89 (0.96-8.69)	0.059
Group III	1.62 (1.07-2.45)	0.023	5.82 (1.97-17.19)	<0.00 1	1.74 (1.12-2.70)	0.014	16.22 (4.21-62.54)	<0.00 1
Anatomic subdivision (ref: Left)								
Right	1.25 (0.66-2.36)	0.499	0.73 (0.31-1.72)	0.473	1.35 (0.70-2.62)	0.366	0.96 (0.36-2.57)	0.931
Bilateral	1.05 (0.64-1.72)	0.838	1.53 (0.69-3.38)	0.291	1.04 (0.63-1.73)	0.873	2.42 (0.96-6.12)	0.062
Age, y (ref: ≤ 60)								
> 60	1.53 (1.10-2.12)	0.011	2.50 (1.26-4.95)	0.009	1.60 (1.13-2.28)	0.009	1.56 (0.76-3.21)	0.226
TNM stage (ref: I/II)								
III	1.13 (0.50-2.56)	0.776	5.51 (2.31-13.15)	<0.00 1	1.10 (0.47-2.60)	0.827	4.76 (1.89-12.01)	0.001
IV	1.04 (0.41-2.63)	0.934	6.62 (2.14-20.46)	0.001	1.25 (0.47-3.28)	0.658	7.38 (1.86-29.25)	0.004
Histologic grade (ref: G1/2)								
G3/4	1.43 (0.86-2.38)	0.173	1.41 (0.71-2.81)	0.329	1.58 (0.93-2.69)	0.094	1.73 (0.65-4.58)	0.269

*Analysis of ovarian cancer patients with complete clinical information.

CI, Confidence interval; HR, Hazard ratio; TCGA, The cancer genome atlas; TNM, Primarytumor, regional lymph node, distant metastasis. Group I: PD-1^low^LAG-3^low^, Group II: PD-1^high^ or LAG-3^high^, Group III: PD-1^high^LAG-3^high^.

### Increased co-expression of PD-1 and LAG-3 proteins was associated with poor OS in our clinical center

**Figure 4:**
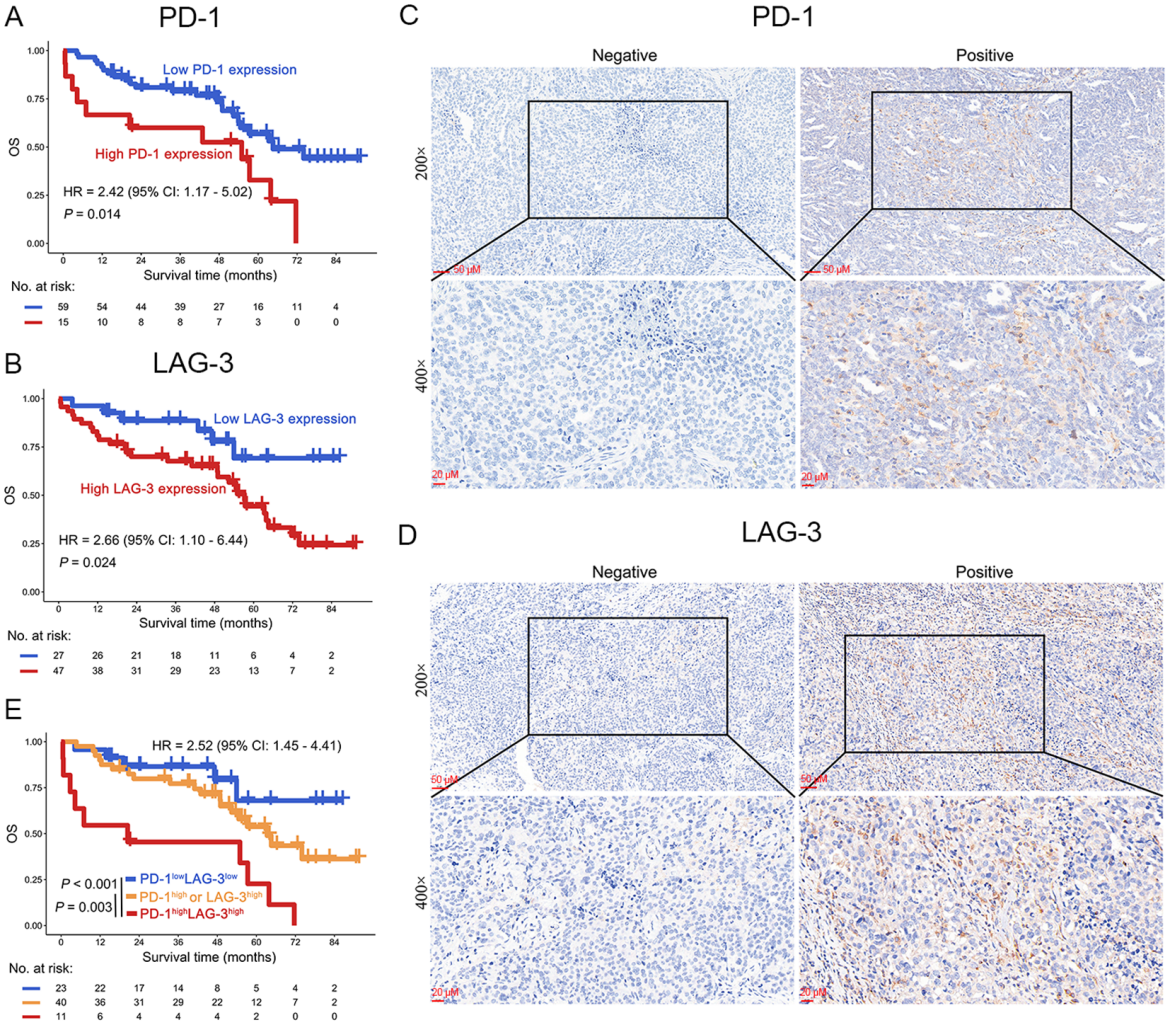
OS analysis of PD-1 and LAG-3 in OC patients in our clinical center. **(A, B)** Protein expression levels of PD-1 **(A)** and LAG-3 **(B)** demonstrated significant associations with OS in OC patients. **(C, D)** Representative immunohistochemical staining micrographs depicting PD-1 **(C)** and LAG-3 **(D)** expression in ovarian tumor tissues. **(E)** Kaplan-Meier curves were plotted based on the different groups of PD-1/LAG-3.

To further validate the results obtained in the TCGA dataset, we investigated the relationship between the protein expression levels of PD-1 and LAG-3 and OS in patients with OC through IHC in our clinical center. The results suggest that high expression of PD-1 was significantly associated with poor OS in patients with OC (HR = 2.42, 95% CI: 1.17-5.02, *P* = 0.014), and this result was found in LAG-3 (HR = 2.66, 95% CI: 1.10-6.44, *P* = 0.024) (**Figures 4A–D**). Critically, co-expression of PD-1 and LAG-3 at the protein level demonstrated superior predictive value for adverse outcomes in patients with OC compared to individual IC biomarkers (group III vs. II: *P* = 0.003; group III vs. I: *P* < 0.001; **Figure 4E**). Compared to the TCGA dataset, the PD-1 and LAG3 combination in the validation set can significantly distinguish patients with favorable, intermediate, and poor prognoses. The above differences may be due to in the validation set, groups 1, 2, and 3 have significant differences in age, anatomic subdivision, TNM stage, histologic grade and histologic type (*P* < 0.001, **Supplementary Table S1**). Notably, further subgroup analysis suggests that elevated PD-1/LAG-3 coexpression significantly correlated with poor OS in patients with serous cystadenocarcinoma (HR = 3.46, 95% CI: 1.66-7.21, *P* < 0.001) (**Supplementary Figure S1A**, **Supplementary Table S1**).

### Risk stratification for patients with OC

**Figure 5:**
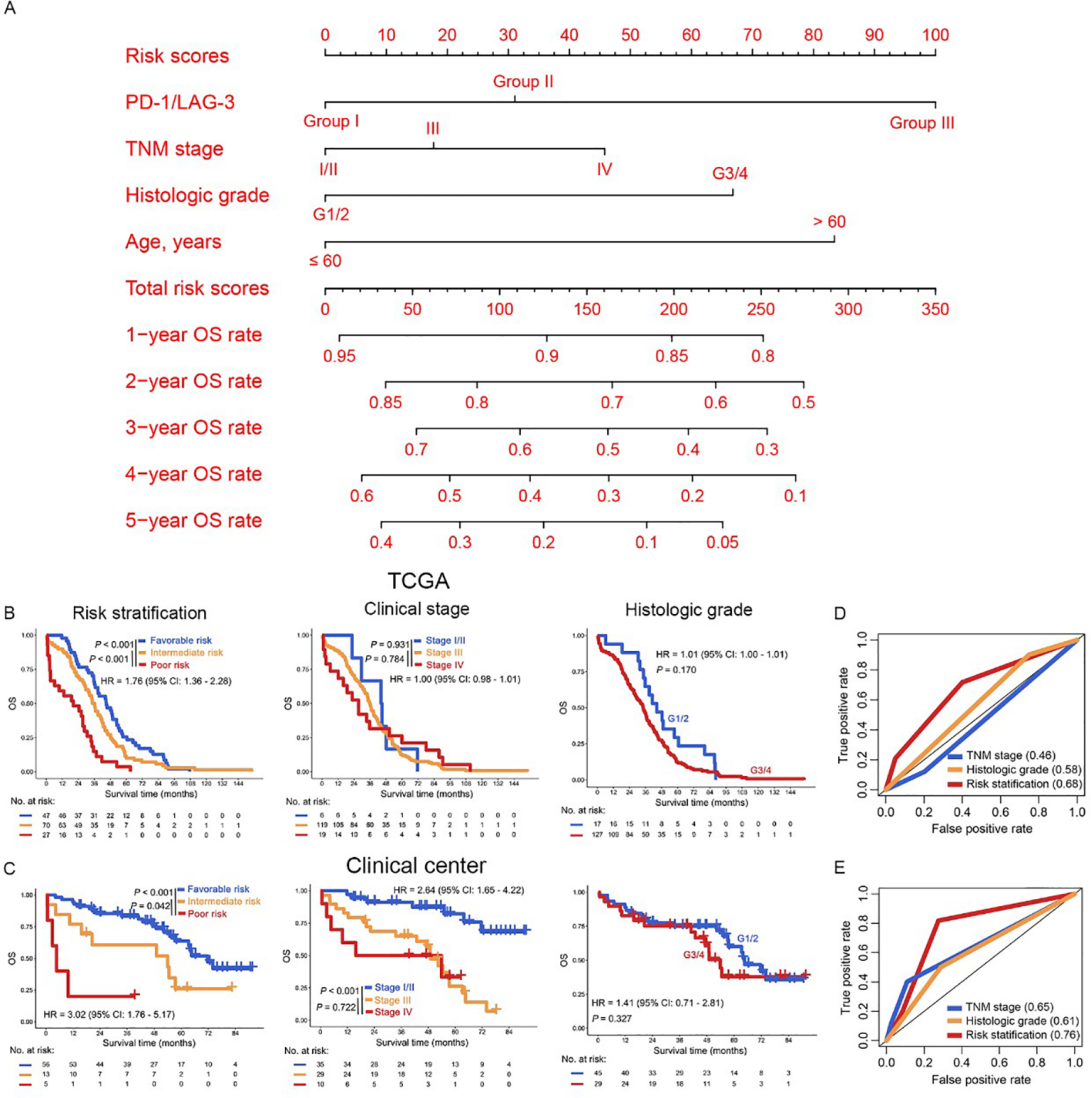
Construction of risk stratification for OC patients. **(A)** The PD-1/LAG3, TNM stage, histologic grade and age were used to construct the nomogram model in the TCGA dataset. **(B, C)** Kaplan Meier curves were plotted according to different subgroups of risk stratification (left panel), clinical stage (middle panel) and histologic grade (right panel) in the TCGA dataset **(B)** and our clinical center **(C)**. **(D, E)** The AUC of risk stratification, clinical stage and histologic grade in the ROC curves were compared in the TCGA dataset **(D)** and our clinical center **(E)**.

Given the pivotal role of risk stratification in directing OC therapeutics, we established an integrative prognostic framework incorporating IC biomarkers and clinicopathological variables. This model significantly improves precision in outcome prediction compared to traditional parameters alone. The multivariable nomogram integrated PD-1/LAG-3 co-expression status with key clinicopathological determinants (TNM stage, histologic grade, age), generating personalized 1- to 5-year OS predictions for patients with OC (**Figure 5A**). Importantly, risk stratification using nomogram-derived cutoffs (132 and 201) delineated three prognostically distinct risk cohorts (favorable, intermediate and poor) in OC. This model demonstrated superior OS discrimination versus conventional TNM staging or histologic grading (**Figure 5B**), with robust external validation in our clinical center (**Figure 5C**), especially in patients with serous cystadenocarcinoma (**Supplementary Figure S1B**, **Supplementary Table S1**). Further ROC curves confirmed that risk stratification derived from the nomogram model predicted the prognosis of OC patients better than TNM staging or historical grading in the both TCGA dataset and our clinical center (**Figures 5D, E**). Therefore, the combined evaluation of PD-1/LAG-3 co-expression patterns and fundamental clinicopathological parameters—TNM stage, histologic grade, and age— significantly augments prognostic discrimination in OC risk assessment frameworks, especially in patients with serous cystadenocarcinoma.

## Discussion

OC remains one of the most aggressive malignancies, and despite advances in treatment, including surgery and platinum-based chemotherapy, the survival rate for patients diagnosed at advanced stages remains alarmingly low (3, 4). The identification of reliable prognostic biomarkers is essential for improving clinical decision-making and patient outcomes. Immunotherapy, particularly ICIs, has shown promise in treating OC (2). However, the clinical efficacy of ICIs in OC has been limited (2, 8, 15). This is largely due to the highly immunosuppressive nature of the OC TME and the absence of reliable biomarkers to predict response and prognosis (23). A significant gap in current research is the lack of systematic analysis of IC co-expression patterns and their prognostic value in OC. In this study, we utilized survival curve analysis to examine data from 147 newly diagnosed OC patients from the TCGA database, alongside 74 clinical tissue samples for validation. Our findings reveal that the co-expression of PD-1 and LAG-3 serves as the optimal combination for predicting poor prognosis in OC patients, especially in patients with serous cystadenocarcinoma. This discovery enables more accurate patient stratification, identifying those who may benefit most from immunotherapy.

Given the limited efficacy of single-agent ICIs, combination therapy has emerged as a promising strategy to overcome immune evasion mechanisms in OC (2, 8, 15, 24). Previous studies have shown that combined blockade of multiple immune checkpoints, such as PD-1, PD-L1, LAG-3, TIGIT, CTLA-4, and TIM3, can improve treatment response and extend OS (2, 25–28). In this study, we found that high expression levels of PD-1 and LAG-3 are significantly associated with OS in OC patients, making them the most promising combination for predicting prognosis. Notably, simultaneous blockade of PD-1 and LAG-3 has demonstrated increased response rates in clinical trials for other malignancies, further supporting the potential of this combination therapy in OC. These findings suggest that dual inhibition of PD-1 and LAG-3 could enhance the anti-tumor immune response and improve the overall efficacy of immunotherapy in OC. This novel discovery lays the groundwork for future studies exploring combination immunotherapy strategies in OC.

The use of TNM staging and pathological grading remains the cornerstone of risk stratification in OC (29, 30). However, these methods have limitations, as they do not fully account for the heterogeneity in patient responses and prognosis (30). In recent years, nomogram-based models have emerged as a promising tool for predicting patient outcomes. Nomograms significantly outperform conventional models (TNM staging, pathological grading) by integrating multivariable predictors into visually interpretable algorithms. They provide individualized risk quantification through point-based scoring systems, enabling precise estimation of clinical outcomes. This approach enhances discriminatory accuracy and calibration versus traditional staging systems. Crucially, nomograms facilitate bedside application without computational tools, allowing dynamic assessment of treatment alternatives (22, 31–33). In this study, we developed a novel risk stratification model incorporating PD-1/LAG3 co-expression, TNM staging, pathological grading, and age. This model significantly improved the prediction of 1–5 year OS rates compared to traditional TNM and pathological grading methods. Patients were classified into favorable, intermediate, and poor-risk subgroups based on this model, which provided more precise prognostic information and highlighted the potential clinical utility of this integrated approach in guiding treatment decisions.

Despite the promising findings of this study, several limitations need to be addressed. First, the validation of PD-1 and LAG-3 co-expression as a prognostic marker was based on clinical tissue samples from a single-center cohort, which may limit the broader applicability of our results. To enhance the generalizability, future studies should involve multi-center cohorts with larger sample sizes to confirm the predictive value of PD-1/LAG-3 co-expression in OC patients. Second, the study did not include cellular and animal model experiments to explore the effects of combined PD-1 and LAG-3 antibody therapy, as well as failed to achieve the validation of protein expression in fresh tissues. Further research using these models is crucial to uncover the underlying mechanisms that drive the enhanced anti-tumor response and to deepen our understanding of how best to implement combination immunotherapy in OC. Finally, limitations arising from the difference between mRNA and protein layers. The TCGA analysis was based on mRNA-level data, but the institutional validation was performed via IHC (protein-level), which was a discrepancy in molecular layers.

In conclusion, we identify the co-expression of PD-1 and LAG-3 as the optimal immune checkpoint combination for predicting prognosis in OC patients.

## Data Availability

The original contributions presented in the study are included in the article/**Supplementary Material**, further inquiries can be directed to the corresponding author/s.

## Supplementary material

Supplementary Table 1 Clinical information of patients with ovarian cancer.

Supplementary Figure 1 OS analysis in patients with serous cystadenocarcinoma in our clinical center. **(A, B)** Kaplan-Meier curves were plotted based on the different groups of PD-1/LAG-3 **(A)** and risk stratification **(B)**. Group I: PD-1^low^LAG-3^low^, Group II: PD-1^high^ or LAG-3^high^, Group III: PD-1^high^LAG-3^high^.
