# Supplementary material for: PD-1 and LAG-3 were optimal combination of immune checkpoints for predicting poor clinical outcomes of patients with ovarian cancer
Source: Front Immunol. 2025 Aug 14;16:1656242. doi: 10.3389/fimmu.2025.1656242 (PMC12390975; doi:10.3389/fimmu.2025.1656242)
Supplement: Supplementary Table 1 — Clinical information of patients with ovarian cancer. [file Presentation1.pdf]

PD-1 and LAG-3 were optimal combination of immune checkpoints for predicting poor clinical outcomes of patients with ovarian cancer

Table S1. Clinical information of patients with ovarian cancer.

Variables	TCGA	Clinical center	*P* value
Number	147	74	
Age, y, mean ± SD	61 ± 11	54 ± 12	<0.001
Anatomic subdivision, n (%)			<0.001
Left	19 (12.9)	36 (48.6)	
Right	19 (12.9)	22 (29.7)	
Bilateral	104 (70.7)	16 (21.6)	
Unknown	5 (0)	0 (0)	
TNM stage, n (%)			<0.001
I	0 (0)	22 (29.7)	
II	6 (4.1)	13 (17.6)	
III	122 (83.0)	29 (39.2)	
IV	19 (12.9)	10 (13.5)	
Histologic grade, n (%)			<0.001
G1	0 (0)	10 (13.5)	
G2	17 (11.6)	35 (47.3)	
G3	126 (85.7)	25 (33.8)	
G4	1 (0.7)	4 (5.4)	
Unknown	3 (2.0)	0 (0)	

Histologic type, n (%)			<0.001
Serous Cystadenocarcinoma	147 (100)	54 (73.0)	
Clear cell carcinoma	0 (0)	20 (27.0)	

SD: Standard deviation; TCGA: The cancer genome atlas; TNM: Primary tumor, regional lymph node, distant metastasis.

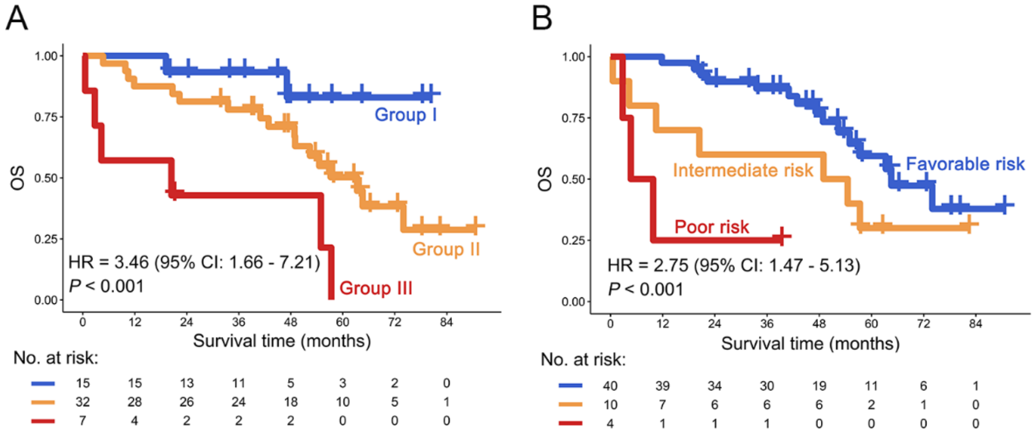

Figure S1. OS analysis in patients with serous cystadenocarcinoma in our clinical center. A-B: Kaplan-Meier curves were plotted based on the different groups of PD-1/LAG-3 (A) and risk stratification (B). Group I: PD-1^{low}LAG-3^{low}, Group II: PD-1^{high} or LAG-3^{high}, Group III: PD-1^{high}LAG-3^{high}.
